# Insulin resistance and obesity, and their association with depression in relatively young people: findings from a large UK birth cohort

**DOI:** 10.1017/S0033291719000308

**Published:** 2020-03

**Authors:** B. I. Perry, G. M. Khandaker, S. Marwaha, A. Thompson, S. Zammit, S. P. Singh, R. Upthegrove

**Affiliations:** 1 Department of Psychiatry, University of Cambridge, Cambridge, England; 2 Cambridgeshire and Peterborough National Health Service Foundation Trust, Cambridge, England; 3 National Institute for Health Research Cambridge Biomedical Research Centre, Cambridge, England; 4 Institute for Mental Health, University of Birmingham, Birmingham, England; 5 Birmingham and Solihull Mental Health Foundation NHS Trust, Birmingham, England; 6 Coventry and Warwickshire Partnership NHS Trust, Coventry, England; 7 Unit of Mental Health and Wellbeing, University of Warwick, Coventry, England; 8 Centre for Academic Mental Health, School of Social and Community Medicine, University of Bristol, Bristol, England; 9 Institute of Psychological Medicine and Clinical Neurosciences, Medical Research Council Centre for Neuropsychiatric Genetics and Genomics, Cardiff University, Cardiff, Wales; 10 Early Intervention Service, Birmingham Women's and Children's NHS Trust, Birmingham, UK

**Keywords:** ALSPAC, depression, inflammation, insulin resistance, obesity, overweight

## Abstract

**Background:**

Depression frequently co-occurs with disorders of glucose and insulin homeostasis (DGIH) and obesity. Low-grade systemic inflammation and lifestyle factors in childhood may predispose to DGIH, obesity and depression. We aim to investigate the cross-sectional and longitudinal associations among DGIH, obesity and depression, and to examine the effect of demographics, lifestyle factors and antecedent low-grade inflammation on such associations in young people.

**Methods:**

Using the Avon Longitudinal Study of Parents and Children birth cohort, we used regression analyses to examine: (1) cross-sectional and (2) longitudinal associations between measures of DGIH [insulin resistance (IR); impaired glucose tolerance] and body mass index (BMI) at ages 9 and 18 years, and depression (depressive symptoms and depressive episode) at age 18 years and (3) whether sociodemographics, lifestyle factors or inflammation [interleukin-6 (IL-6) at age 9 years] confounded any such associations.

**Results:**

We included 3208 participants. At age 18 years, IR and BMI were positively associated with depression. These associations may be explained by sociodemographic and lifestyle factors. There were no longitudinal associations between DGIH/BMI and depression, and adjustment for IL-6 and C-reactive protein did not attenuate associations between IR/BMI and depression; however, the longitudinal analyses may have been underpowered.

**Conclusions:**

Young people with depression show evidence of DGIH and raised BMI, which may be related to sociodemographic and lifestyle effects such as deprivation, smoking, ethnicity and gender. In future, studies with larger samples are required to confirm this. Preventative strategies for the poorer physical health outcomes associated with depression should focus on malleable lifestyle factors.

## Introduction

Depression in 10–24 year olds is a leading cause of disease burden throughout the world (Gore *et al*., 2011). An important aspect of this burden is the co-occurrence of disorders of glucose and insulin homeostasis (DGIH) [type-2 diabetes mellitus (T2DM) and prediabetes] and obesity (Roy and Lloyd, 2012; Vancampfort *et al*., 2016). This may be a consequence of disease-related factors such as symptomatology [e.g. appetite disturbance and decreased physical activity (Lysy *et al*., 2008; Vancampfort *et al*., 2017)], increased rates of smoking (Katon *et al*., 2004), alcohol use (Tann *et al*., 2007), an unhealthy diet (Firth *et al*., 2018) and also sociodemographic risk factors such as either male (Nichols and Brown, 2003; Ali *et al*., 2006; Ding *et al*., 2006; Timonen *et al*., 2006; Perreault *et al*., 2008; Menke *et al*., 2014) or female (Anderson *et al*., 2001; Blazer *et al*., 2002; Nichols and Brown, 2003; Ali *et al*., 2006; Lloyd *et al*., 2018) sex, non-white European race/ethnic group (Blazer *et al*., 2002; Li *et al*., 2008; Dagenais *et al*., 2016; Mangurian *et al*., 2018) and lower social class or adversity (Everson *et al*., 2002; Tamayo *et al*., 2010; Pisto *et al*., 2014).

Another postulated mechanism is that depression, DGIH and obesity are intrinsically linked beyond the above via common antecedent inflammatory processes. Raised interleukin-6 (IL-6) and tumour necrosis factor alpha are antecedent to insulin resistance (IR) (Pickup, 2004; Belgardt *et al*., 2010), and subsequently T2DM and obesity (DeFronzo and Ferrannini, 1991; Dandona *et al*., 2004; Rader, 2007). A recent genome-wide association study (Milaneschi *et al*., 2017) found that common genetic variants for body mass index (BMI) and C-reactive protein (CRP) show overlap with gene variants associated with depression. In addition, inflammation may be prospectively linked to depression in young people, with longitudinal cohort-based research finding raised levels of IL-6 during childhood to be associated with future depressive symptoms and diagnosis of depression at age 18 years, which persisted after controlling for BMI, social class, and childhood psychological and behavioural problems preceding IL-6 measurement (Khandaker *et al*., 2014; Khandaker *et al*., 2018*a*, 2018*b*). This may relate to antecedent stressful events (Slopen *et al*., 2013). A recent large meta-analysis has added to these findings, with cytokines including IL-6 marked as part of a potential chemokine/cytokine profile associated with depression (Köhler *et al*., 2017).

The finding that low-grade systemic inflammation appears antecedent to DGIH, obesity and depression may be evidence of a common biological pathway that begins with an inflammatory response. Research examining the association among DGIH, obesity and depression, particularly longitudinally, in a sample of relatively young people who are less affected by years of illness, is scarce. It is nonetheless an important extension of the current literature as the findings may promote earlier and closer monitoring of metabolic and inflammatory function in young people with depression. It may in addition further our pathophysiological understanding of the multi-systemic nature of depression and suggest possible preventative therapeutic targets (Insel and Charney, 2003).

Using longitudinal population-based data, we tested the hypothesis that even relatively young people with depression may display early signs of DGIH or obesity. We tested cross-sectional (age 18 years) and longitudinal (age 9 and 18 years) associations among DGIH, obesity and depression. We hypothesised that early signs of DGIH might be explained by shared inflammatory processes, or demographic/lifestyle factors. We tested this by assessing for any attenuation (confounding) effect of either demographic, lifestyle or inflammatory measures on associations among DGIH, obesity and depression.

## Methods

### Description of cohort and sample selection

The Avon Longitudinal Study of Parents and Children (ALSPAC) birth cohort (Boyd *et al*., 2013; Fraser *et al*., 2013) comprises 14 062 live births from mothers residing in (former) Avon County, southwest England, with expected dates of delivery between April 1991 and December 1992. Please note that the study website contains details of all the data that are available through a fully searchable data dictionary and variable search tool (http://www.bristol.ac.uk/alspac/researchers/our-data/). Parents completed regular postal questionnaires about all aspects of their child's health and development from birth. From age 7 years, the children attended an annual assessment clinic during which they participated in various face-to-face interviews and physical tests. In an attempt to boost study numbers, further phases of recruitment took place after the age of 7 years, leading to an additional 713 participants recruited by age 18.

We first selected all participants with a measure for the outcome (*n* = 4563) and removed participants with CRP > 10 (Khandaker *et al*., 2014), to minimise the potential confounding effect of ongoing or recent inflammatory disease/infection on our results, leaving 3208 participants. For cross-sectional analyses, 2231 participants had complete data on all exposure and confounder variables. For longitudinal analyses, 331 participants had complete data on all exposure and confounder variables. The lower sample with complete data for longitudinal analyses is explained by age 9 glycaemic data being derived from a smaller ALSPAC sub-study (Ong *et al*., 2004). Complete-case analysis is presented in online Supplementary Tables 1 and 2. See statistical methods for our means of addressing missing data.

The study received ethics approval from the ALSPAC Ethics and Law Committee and local research ethics committees. All participants provided written informed consent.

## Outcome measures

### Depressive symptoms/episode at age 18 years

Depression was measured using the Clinical Interview Schedule-Revised (CIS-R), a widely used standardised self-assessment tool for measuring depression and anxiety in community samples (Lewis *et al*., 1992). It includes symptoms of depression based on *International Statistical Classification of Diseases, 10th Revision* (ICD-10) criteria, and gives a total depression score of 0–21 comprising symptom scores for depression, depressive thoughts, fatigue, concentration and sleep problems. Our primary outcome measure was the continuous CIS-R depression score (Khandaker *et al*., 2014). As a secondary outcome, we created a binary variable ‘depressive episode’. This consisted of participants meeting ICD-10 criteria for a depressive episode (mild/moderate/severe) (F32.0/F32.1/F32.2), as has been used previously (Bowes *et al*., 2015; Davies *et al*., 2016; Quarini *et al*., 2016).

## Exposures

### Fasting plasma glucose, fasting insulin, glucose tolerance

We used the biochemical measurements of fasting plasma glucose (FPG) and fasting insulin (FI) (ages 9 and 18 years), and 2-h glucose tolerance (2hrGT) (age 9 years). The age 9 glycaemic data were derived from a smaller ‘Before Breakfast Study’ sub-study (Ong *et al*., 2004). At both ages 9 and 18 years, fasting samples were taken at 0900 after a 10-h fast (water only). The 2hrGT test was obtained following the above fasting procedure, with the addition of a 75 g oral bolus of sugary syrup at 0900, with blood being sampled 2 h later. Blood samples were immediately spun and frozen at −80 °C.

IR was calculated as a continuous measure from FPG and FI by using the computerised, updated version of the homoeostatic measurement for insulin resistance (HOMA_2_) (Levy *et al*., 1998). The algorithm generates a relatively precise measurement of IR taking into account variations in hepatic and peripheral glucose resistance, increases in the insulin secretion curve for plasma glucose concentrations above 10 mmol/L (180 mg/dL) and the contribution of circulating proinsulin (Levy *et al*., 1998). We did not include a binary measure for clinical IR due to the known variation in HOMA_2_ score between populations (Wallace *et al*., 2004) thus ascertaining a clinical ‘cut-off’ may be problematic.

### BMI

BMI was calculated from clinic data in the ALSPAC cohort, from measurements of height (m) and weight (kg). We used data collected at ages 9 and 18 years.

### Demographic confounders

We adjusted for paternal social class at birth (questionnaire data, categorical) and maternal education (questionnaire data, categorical) as proxies of participant social class and potential adversity, sex (clinic data, categorical), ethnicity (questionnaire data, categorical) and maternal Edinburgh Post-Natal Depression Score (EPDS) at 8-week post-partum (questionnaire data, continuous).

### Lifestyle confounders

For analyses on DGIH we adjusted for BMI (clinic data, continuous, ages 9 and 18 years as per the exposure), smoking (questionnaire data, categorical), cortisol levels [age 9 only, continuous, clinic data from BBS sub-study (Ong *et al*., 2004)], physical activity (questionnaire data on average frequency of physical activity/exercise per week in the last year, categorical) and alcohol use (questionnaire data on average frequency of use, categorical). For BMI analyses, we used the same adjustments, however with HOMA_2_ (as previously described, continuous) in place of BMI.

### Inflammatory confounders

We adjusted for IL-6 (age 9 years) and CRP (age 18 years). IL-6 was not available at age 18 years. Blood samples were collected from non-fasting participants (age 9 years), and fasting participants (age 18 years) and were immediately spun and frozen at −80 °C. IL-6 was measured by enzyme-linked immunosorbent assay (R&D Systems), and high-sensitivity CRP (hs-CRP) was measured by automated particle-enhanced immunoturbidimetric assay (Roche). All inter-assay coefficients of variation were less than 5%.

## Statistical analysis

Biomarker values that were non-normally distributed (all except for FPG) were natural log-transformed. Resultant variables, alongside the continuous outcome ‘total depression score’ were standardised (*Z*-transformed) so the statistical estimations represent the increase in risk of depressive symptoms per s.d. increase in exposure. We completed tests for multi-collinearity of exposures/confounders in a linear regression model. The variance inflation factor for all covariates was between 1.01 and 1.11, suggesting minimal multi-collinearity. Adjustments were added using the enter method of multiple regression. All statistical analysis was performed using IBM SPSS 24.0.

### Aims 1 and 2: cross-sectional and longitudinal relationships between DGIH/BMI (ages 9 and 18 years) and depression (age 18 years)

We completed cross-sectional linear and logistic regression analyses which examined the relationship between markers of DGIH/BMI and depressive symptoms/depressive episode (age 18 years). We completed longitudinal linear and logistic regression analyses which examined the relationship between markers of DGIH/BMI (age 9 years) and depressive symptoms/episode (age 18 years). Regression coefficients and 95% confidence intervals (95% CIs) were calculated per s.d. increase in the continuous ‘total depression score’ outcome, per s.d. increase in exposure, using linear regression. Odds ratios (ORs) and 95% CIs for the categorical depressive episode outcome, per s.d. increase in exposure, were estimated using logistic regression. Quadratic terms were created separately for all exposures and entered into a logistic regression model to simulate curvilinear regression, to test the linearity of relationships between exposures and depression; these data are only shown where there was evidence of a non-linear relationship.

### Aim 3: adjusting for demographic factors, lifestyle factors and inflammation

We performed adjustments using linear and logistic regression as described above. First, we adjusted for the demographic, lifestyle and inflammation factors listed above, separately. Second, we completed a total adjustment model including all potential confounders together.

### Missing data

Missing data for exposures and confounders were present in 30% of cases for cross-sectional analyses, and 90% of cases for longitudinal analyses. Due to the substantial amount of missing data in longitudinal analyses [which may be related to the age 9 glycaemic data being derived from a smaller ALSPAC sub-study (Ong *et al*., 2004)], we longitudinally analysed only complete cases (Lee and Huber, 2011). For our cross-sectional analyses, Little's Missing Completely at Random (MCAR) test (*p* = 0.008) indicated that the data were not MCAR. We then used the missing value analysis function of SPSS to perform separate variance independent *t* tests (continuous variables) and χ^2^ tests (categorical variables) to check the missing at random (MAR) assumption. Each variable returned significance (*p* < 0.05) with at least one other included variable, indicating that missingness was correlated with another variable in the model, suggesting the missing data met the MAR assumption.

We completed multiple imputation (MI) using the fully conditional Markov chain Monte Carlo method, for all exposure and confounder variables, plus axillary continuous variables that were indicators of missingness in the population. The selected axillary variables included age 9 biochemical data (high-density lipoprotein, low-density lipoprotein, triglycerides), as well as birthweight and gestational age. As missing data were present in 30% of cases, we used 30 imputations as recommended (White *et al*., 2011). Complete case analysis for the cross-sectional analyses is presented in online Supplementary Tables 1 and 2.

## Results

Following imputation for exposure and confounder variables, our total sample was 3208 participants. The mean depression score in the imputed sample was 3.09; range 0–21 (complete cases 3.10; range 0–21). The number of participants meeting criteria for a depressive episode at age 18 was *n* = 227 (7%) (complete cases *n* = 179; 8%). Table 1 shows the sample clinical and biomarker characteristics at age 18.

**Table 1.** Baseline characteristics of sample

Characteristic	All sample	Depressive episode	No depressive episode	Test statistic^a^ *p*-value
Participants, *n* (% total)	3208 (100)	227 (7)	2981 (93)	–
Male sex, *n* (column %)	1553 (48)	62 (27)	1491 (50)	9.167; *p* = 0.002
Smoking at 18 years, *n* (column %) (current)	231 (10)	27 (11)	220 (7)	7.535; *p* = 0.006
Maternal EPDS score^b^, mean (s.d.)	5.89 (4.65)	5.78 (4.65)	5.98 (4.69)	0.476 *p* = 0.322
Maternal education (A-levels or above), *n* (column %)	1116 (35)	74 (33)	1049 (35)	0.267 *p* = 0.096
Alcohol use^c^ (greater than once per week), *n* (column %)	1320 (41)	132 (58)	1070 (36)	8.756 *p* = 0.024
Physical activity^d^ (greater than once per week), *n* (column %)	2213 (69)	78 (35)	2337 (78)	9.967 *p* < 0.001
Father's social class, *n* (column %)				4.235; *p* = 0.055
I	186 (6)	15 (7)	184 (6)	
II	1119 (35)	73 (32)	1077 (36)	
III – non-manual	1257 (39)	87 (38)	1145 (38)	
III – manual	96 (3)	7 (3)	85 (3)	
IV	465 (14)	36 (16)	394 (13)	
V	95 (3)	9 (4)	96 (3)	
White British ethnicity, *n* (column %)	3143 (98)	221 (97)	3175 (99)	0.243; *p* = 0.588
BMI at 18 years, mean (s.d.), kg/m^2^	22.68 (3.81)	23.06 (5.38)	22.65 (3.79)	8.141; *p* = 0.330
BMI at 9 years, mean (s.d.), kg/m^2^	17.54 (2.53)	17.88 (2.23)	17.51 (2.46)	1.967; *p* = 0.223
FPG at 18 years, mean (s.d.), mmol/L	5.04 (0.59)	4.95 (0.76)	5.09 (0.48)	0.044; *p* = 0.389
HOMA_2_ at 18 years, mean (s.d.)	0.92 (0.73)	1.32 (0.55)	0.87 (0.44)	3.860; *p* = 0.035
CRP at 18 years, mean (s.d.), mg/L	1.11 (1.41)	1.20 (1.05)	1.08 (1.43)	0.423; *p* = 0.701
IL-6 at 9 years, mean (s.d.), pg/mL	1.21 (0.78)	1.27 (1.58)	1.18 (1.52)	7.687; *p* = 0.004
CRP at 9 years, mean (s.d.), pg/mL	0.68 (2.52)	0.76 (1.92)	0.65 (3.54)	4.723; *p* = 0.226

a Categorical variables (sex, social class, ethnicity, smoking) were compared using the χ^2^ test, normally distributed continuous variables (FPG, birthweight, gestational age) were compared using the two tailed *t* test; non-normally distributed continuous variables (HOMA_2_, CRP, IL-6, BMI) were compared using the Mann–Whitney *U* test.

b Maternal EPDS score recorded at 8 week post-partum.

c Frequency participant has had drinks containing alcohol.

d Physical activity corresponded to frequency respondent engaged in going to gym, brisk walking or any sports activity during the past year

### Aim 1: cross-sectional association between DGIH/BMI and depression (age 18 years)

In the unadjusted analyses; HOMA_2_, FI and BMI were positively associated with depressive symptoms at age 18 years [*β* = 0.04 (95% CI 0.03–0.30) *p* = 0.02; *β* = 0.05 (95% CI 0.03–0.33) *p* = 0.01; *β* = 0.03 (95% CI 0.01–0.08) *p* = 0.04 respectively]; FPG was negatively associated with depressive symptoms [*β* = −0.05 (95% CI −0.36 to −0.02) *p* = 0.01]. See Table 2. The results from our complete case analysis were broadly similar. See online Supplementary Table 1.

**Table 2.** Cross-sectional association between DGIH/BMI and depressive symptoms (age 18)

Predictor	Regression coefficient (95% CI) for depressive symptoms										
	Adjustments										
	Unadjusted model			Demographic adjustments (sex, birth social class, ethnicity, maternal education, maternal EPDS score)		Lifestyle adjustments (BMI^a^, HOMA_2_^b^, smoking, alcohol use physical activity)		Immune adjustments [IL-6 (9 years), CRP (18 years)]		Complete model (all adjustments)	
Depressive symptoms											
	*n*	*β* (95% CI)	*p*	*β* (95% CI)	*p*	*β* (95% CI)	*p*	*β* (95% CI)	*p*	*β* (95% CI)	*p*
HOMA_2_	3208	0.04 (0.02–0.30)	0.023*	0.05 (−0.07–0.21)	0.336	0.04 (−0.09 to 0.18)	0.100	0.04 (0.01–0.31)	0.031*	0.03 (−0.01 to 0.18)	0.638
FPG	3208	−0.05 (−0.36 to −0.02)	0.014*	−0.02 (−0.17–0.13)	0.816	−0.04 (−0.30 to 0.07)	0.067	−0.04 (−0.32 to −0.02)	0.021*	−0.04 (−0.18 to 0.11)	0.636
Fasting insulin	3208	0.05 (0.03–0.33)	0.013*	0.06 (−0.06–0.13)	0.282	0.05 (−0.02 to 0.12)	0.059	0.04 (0.01–0.17)	0.023*	0.05 (−0.11 to 0.20)	0.553
BMI	3208	0.03 (0.01–0.08)	0.046*	0.03 (−0.01–0.07)	0.144	0.03 (−0.01 to 0.07)	0.062	0.03 (0.01–0.09)	0.031*	0.02 (−0.02 to 0.06)	0.240

a Not adjusted for in BMI analysis.

b Not adjusted for in HOMA/FPG/FI analysis.

*Indicates *p* < 0.05.

In the unadjusted analyses, both HOMA_2_ and FI were associated with the categorical depressive episode [OR 1.14 (95% CI 1.01–1.31) *p* = 0.04 and OR 1.16 (95% CI 1.01–1.33) *p* = 0.03 respectively]. See Table 3. The results are similar to those for our complete case analysis. See online Supplementary Table 2.

**Table 3.** Cross-sectional associations between DGIH/BMI and depressive episode (age 18 years)

Predictor		Odds ratio (95% CI) for depressive episode										
		Adjustments										
Depressive episode		Unadjusted model			Demographic adjustments (sex, birth social class, ethnicity, maternal education, maternal EPDS score)		Lifestyle adjustments (BMI^a^, HOMA_2_^b^, smoking, alcohol use, physical activity)		Immune adjustments [IL-6 (9 years), CRP (18 years)]		Complete model (all adjustments)	
	*N* outcome	*n*	OR (95% CI)	*p*	OR (95% CI)	*p*	OR (95% CI)	*p*	OR (95% CI)	*p*	OR (95% CI)	*p*
HOMA_2_	227	3208	1.14 (1.01–1.31)	0.047*	1.07 (0.97–1.24)	0.296	1.12 (0.97–1.30)	0.132	1.15 (1.01–1.32)	0.042*	1.05 (0.91–1.23)	0.498
FPG	227	3208	0.90 (0.77–1.05)	0.174	1.02 (0.88–1.18)	0.787	0.89 (0.76–1.03)	0.115	0.89 (0.77–1.05)	0.165	1.00 (0.87–1.17)	0.959
FI	227	3208	1.16 (1.01–1.33)	0.034*	1.09 (0.94–1.25)	0.269	1.13 (0.98–1.32)	0.098	1.16 (1.02–1.34)	0.029*	1.06 (0.91–1.24)	0.794
BMI	227	3208	1.02 (0.99–1.06)	0.138	1.02 (0.98–1.05)	0.305	1.01 (0.98–1.05)	0.486	1.03 (0.99–1.07)	0.107	1.02 (0.98–1.06)	0.368

a Not adjusted for in BMI analysis.

b Not adjusted for in HOMA/FPG/FI analysis.

*Indicates *p* < 0.05.

### Aim 2: longitudinal association between DGIH/BMI (age 9 years) and depressive symptoms/episode (age 18 years)

Our longitudinal analysis of DGIH included 399 participants, and for BMI 2571 participants. There were no evident longitudinal associations between DGIH/BMI at age 9 years and depressive symptoms/episode at age 18. See Tables 4 and 5.

**Table 4.** Longitudinal association between DGIH/BMI (age 9) and depressive symptoms (age 18 years)

Predictor	Regression co-efficient (95% CI) for depressive symptoms (age 18 years)										
	Adjustments										
	Unadjusted model			Demographic adjustments (sex, birth social class, ethnicity, maternal education, maternal EPDS score)		Lifestyle adjustments (BMI^a^, HOMA_2_^b^, physical activity)		Immune adjustments [IL-6 (9 years)]		Complete model (all adjustments)	
Depressive symptoms (18 years)											
	*n*	*β* (95% CI)	*p*	*β* (95% CI)	*p*	*β* (95% CI)	*p*	*β* (95% CI)	*p*	*β* (95% CI)	*p*
2hrGT (age 9 years)	399	0.07 (−0.33 to 0.34)	0.172	0.01 (−0.36 to 0.37)	0.980	0.06 (−0.26 to 0.46)	0.563	0.04 (−0.26 to 0.23)	0.595	0.01 (−0.36 to 0.37)	0.980
HOMA_2_ (age 9 years)	399	0.02 (−0.28 to 0.39)	0.739	0.01 (−0.24 to 0.27)	0.917	0.01 (−0.25 to 0.27)	0.954	0.01 (−0.25 to 0.27)	0.942	0.01 (−0.25 to 0.26)	0.972
FPG (age 9 years)	399	0.02 (−0.28 to 0.39)	0.750	0.03 (−0.31 to 0.44)	0.878	0.02 (−0.14 to 0.44)	0.270	0.02 (−0.15 to 0.44)	0.276	0.02 (−0.28 to 0.40)	0.801
FI (age 9 years)	399	0.07 (−0.10 to 0.57)	0.172	0.05 (−0.21 to 0.19)	0.768	0.01 (−0.25 to 0.26)	0.992	0.01 (−0.24 to 0.27)	0.928	−0.01 (−0.25 to 0.24)	0.949
BMI (age 9 years)	2571	0.04 (−0.04 to 0.10)	0.054	0.03 (−0.02 to 0.09)	0.247	0.05 (−0.02 to 0.11)	0.065	0.04 (−0.02 to 0.10)	0.154	0.02 (−0.03 to 0.08)	0.416

a Not adjusted for in BMI analysis.

b Not adjusted for in HOMA/FPG/FI analysis.

*Indicates *p* < 0.05.

**Table 5.** Longitudinal association between DGIH/BMI (age 9) and depressive episode (age 18 years)

Predictor		Odds ratio (95% CI) for depressive episode (age 18 years)										
		Adjustments										
Depressive episode (18 years)	*n* outcome	Unadjusted model			Demographic adjustments (sex, birth social class, ethnicity, maternal education, maternal EPDS score)		Lifestyle adjustments (BMI^a^, HOMA_2_^b^, physical activity)		Immune adjustments [IL-6 (9 years)]		Complete model (all adjustments)	
		*n*	OR (95% CI)	*p*	OR (95% CI)	*p*	OR (95% CI)	*p*	OR (95% CI)	*p*	OR (95% CI)	*p*
2hrGT (age 9 years)	23	313	1.21 (0.95–1.54)	0.235	0.98 (0.73–1.31)	0.891	0.91 (0.60–1.38)	0.641	0.91 (0.60–1.39)	0.651	0.85 (0.55–1.30)	0.427
HOMA_2_ (age 9 years)	23	313	1.16 (0.86–1.20)	0.387	1.14 (0.75–1.72)	0.538	0.98 (0.74–1.31)	0.896	0.98 (0.74–1.30)	0.893	0.98 (0.83–1.31)	0.977
FPG (age 9 years)	23	313	1.03 (0.90–1.07)	0.446	0.85 (0.55–1.30)	0.442	1.08 (0.73–1.61)	0.693	1.05 (0.69–1.58)	0.830	1.19 (0.78–1.84)	0.678
FI (age 9 years)	23	313	1.10 (0.90–1.21)	0.250	1.01 (0.75–1.34)	0.971	1.01 (0.75–1.33)	0.995	1.02 (0.77–1.34)	0.916	0.97 (0.74–1.33)	0.916
BMI (age 9 years)	170	2571	1.23 (0.76–1.32)	0.687	1.04 (0.98–1.10)	0.127	1.05 (0.98–1.11)	0.058	1.06 (0.97–1.12)	0.060	1.05 (0.98–1.12)	0.070

a Not adjusted for in BMI analysis.

b Not adjusted for in HOMA/FPG/FI analysis.

*Indicates *p* < 0.05.

### Aim 3: adjusting for confounders

As shown in Tables 2–5, after adjustments for demographic and lifestyle factors the point estimates did not change considerably but the 95% CIs widened to include the null. Adjustment for immune markers did not significantly alter the unadjusted associations. Following adjustment for all confounder variables in one model, there were no significant associations. Results from the complete case analysis are broadly similar. See online Supplementary Tables 1 and 2.

## Discussion

In this study, we first tested the cross-sectional associations between DGIH/BMI and depression in a sample of young people who may have been less affected by years of illness, before and after adjustments for potential demographic, lifestyle and immune confounders. We then used longitudinal analysis to test the direction of association between these factors. To our knowledge, this is one of the first analyses of detailed longitudinal associations among DGIH, BMI, inflammation and depression, in a relatively young sample, albeit the sample size for some of the analyses was relatively small. We present several findings of note.

We found that the broadest marker of glycaemic function, FPG, was negatively associated with depressive symptoms at age 18 years. In addition, more sensitive markers of pre-clinical glucose dysregulation (FI, HOMA_2_) were positively associated with depression at age 18 years. IR in combination with low FPG shows biological plausibility; FPG can present low-normal in early IR, in response to the IR phenotype of increased insulin secretion thus increased intracellular glucose uptake (Ensling *et al*., 2011). These associations were not significant following demographic and lifestyle adjustments suggesting that the metabolic dysfunction present in depression may be attributable to sociodemographic and lifestyle factors. Interestingly however, whilst the CIs became larger (and included the null) after confounding adjustments, the point estimates did not change substantially. Research conducted on larger samples of depressed patients would therefore be appropriate to increase statistical power, to further test these findings.

Our findings differ from previous longitudinal research from the Northern Finland Birth Cohort (NFBC) (Timonen *et al*., 2006), which found IR to be cross-sectionally associated with depressive symptoms even after adjustments for similar confounders. However, in that study, a different mathematical method, the Qualitative Insulin Sensitivity Check Index (QUICKI) (Katz *et al*., 2000) was used to measure IR. The QUICKI is limited in being blind to several important physiological aspects of glucose homoeostasis; and being calibrated to an aged insulin assay (Wallace *et al*., 2004). We used the computerised, updated HOMA_2_ model that addresses the shortcomings of QUICKI and other early models. Additionally, the participants in the Finnish study were older (age 31 years) thus the potential for confounding by the potential chronic lifestyle factors of depression was increased. Another previous study using NFBC data found no association between depression and the wider metabolic syndrome as a whole, after controlling for similar adjustments at age 31 years (Herva *et al*., 2006). The metabolic syndrome classification may be less sensitive to early metabolic dysfunction than measures of IR. For example, a smaller cross-sectional study from Taiwan of 323 participants (mean age of 19.5 years) found no association between depression and the metabolic syndrome as a whole, but did find associations with specific elements of metabolic dysfunction such as BMI and hypertension, though the associations attenuated following adjustments (Lin *et al*., 2014). Whilst this latter study was relatively small, the results are in line with ours. Another study of young adults from the USA found depression to be associated with the metabolic syndrome at age 30 years (Kinder *et al*., 2004).

We found no associations longitudinally between age 9 markers of DGIH/BMI and later development of depressive symptoms/episode by age 18 years. However, our longitudinal analyses of glycaemic function were susceptible to reduced statistical power due to the smaller sample that underwent glycaemic testing at age 9 years in the cohort. Therefore, since the number of depression events in the longitudinal analyses of glycaemic function was relatively small, the corresponding demographic and lifestyle adjustment models may have been susceptible to model overfit (Peduzzi *et al*., 1996), limiting the generalisability of these results. Results for the longitudinal analyses should therefore be interpreted with caution.

Taken together, our results suggest that the sociodemographic and lifestyle features of the depressive syndrome such as gender, ethnicity, paternal social class, smoking, alcohol use and physical activity levels may be driving the known associations between T2DM, obesity and depression. Nonetheless, sensitive metabolic changes are apparent from a relatively early age. This is an important finding. Whilst the demographic confounders we adjusted for are fixed, the lifestyle confounders we adjusted for may be malleable. For that reason, our results demonstrate the crucial importance for even relatively young patients diagnosed with depression to receive a full and comprehensive assessment of metabolic function. Encouragement and importance should be placed on encouraging and incentivising positive lifestyle changes, such as smoking cessation and reducing alcohol intake. Interestingly, other relevant lifestyle changes such as encouraging a healthy diet and regular exercise show some evidence for having intrinsic mood-boosting properties (Jacka *et al*., 2010; Cooney *et al*., 2014).

### Strengths and limitations

The ALSPAC cohort provided a relatively large sample size in which to conduct analyses and we were able to consider detailed potential confounders including current/recent inflammation, alcohol use, BMI (in IR analyses), IR (in BMI analyses), smoking, physical activity, maternal post-natal EPDS score, paternal social class, ethnicity and sex. We attempted to reduce bias and increase statistical power (Dong and Peng, 2013) by using the MI method to account for missing data where possible.

However, there are several limitations that should be considered. Firstly, we have put emphasis on the effects of lifestyle on glycaemic and anthropometric parameters, however the lifestyle data we collected in our analyses was mostly collected via self-report questionnaires. Self-report questionnaire data on lifestyle factors can be limited in its validity and reliability, for reasons such as social desirability or recall bias (Sallis and Saelens, 2000; Del Boca and Darkes, 2003; Shipton *et al*., 2009). ALSPAC does have a quantitative measure for physical activity at age 18 years. Participants were invited to take part in a week-long wrist accelerometer study, however, the sample size that were able to provide full data on this study was much smaller than data available for the self-report measure; participants were asked to remove the accelerometer for certain types of exercise; and, we felt that data collected in this manner may be susceptible to the Hawthorne effect. Nonetheless, the limitations of using self-report data for lifestyle parameters should be taken into account when interpreting our findings.

Due to the significant amount of missing data in our longitudinal analyses, imputing such missing data may have led to both to selection bias and to type-II statistical error (Lee and Huber, 2011). It is therefore likely our longitudinal analyses, particularly of glycaemic function, are underpowered. In such analyses, models including demographic or lifestyle adjustments are likely to be overfit, limiting their generalisability. Our longitudinal findings should therefore be interpreted with caution. Selection bias is also a possibility since not all ALSPAC participants attended voluntary CIS-R assessment at age 18. In addition, we have used paternal social class at birth as a proxy of social class and potential adversity of the participant and these suppositions may be open to challenge. Furthermore, whilst most biochemical tests were sampled in the fasting state, age 9 inflammatory markers were sampled in the non-fasting state, which may increase measurement error. Measurement error can introduce a bias towards the null, so the results for IL-6 may be underestimated. Also, we have examined an *a priori* hypothesis based upon potential biological plausibility involving immune dysfunction upstream of both DGIH and BMI. However, obesity is known to be a pro-inflammatory state itself (Jung and Choi, 2014), thus reverse causality may be a possibility. Future research may seek to take this into account. Additionally, whilst we restricted our analyses to participants with CRP < 10 mg/L to account for chronic/acute infection/inflammatory illness, we were unable to ascertain whether included participants were in receipt of immune-modulating medications. Finally, whilst we included data for IL-6 and CRP, future analyses may seek to examine additional circulating markers of innate and adaptive immune response.

### Implications and future directions

Our findings have implications both in the assessment and management of patients who present with symptoms of depression. We found that even at the relatively young age of 18 years, depression is associated with DGIH and raised BMI. That the metabolic associations with depression can occur at such an early phase of a potentially chronic course of depression is significant and underlines the need for swift and comprehensive assessment and management of metabolic risk factors in people that present with depression. Our findings may provide impetus for the monitoring of more sensitive measures of metabolic function in people first presenting with depression, since the elements that make up the ‘metabolic syndrome’, of which IR and BMI are a part, are by definition reversible (Alberti *et al*., 2005), and therefore early intervention may help to attenuate the significant morbidity (Goldney *et al*., 2004) and socioeconomic cost (Molosankwe *et al*., 2012) associated with comorbid depression and metabolic dysfunction. Taken together with previous research (Khandaker *et al*., 2014; Miller and Raison, 2016; Khandaker *et al*., 2018*a*) which suggests that immune dysfunction could be a target for prevention and treatment of depression, our findings may suggest that other factors also play an important role in increasing the physical health burden associated with depression. Impetus should be placed on encouraging healthy lifestyles such as with a healthy diet and exercise, which have both shown to be beneficial in improving depression (Schuch *et al*., 2016*a*, 2016*b*; Teasdale *et al*., 2017).

Future research should seek to examine associations between young adults with depression and measures of dyslipidaemia, which may also be relevant (Parekh *et al*., 2017), and should seek to address whether improved recognition and interventions for modifiable lifestyle factors in the early treatment of depression may result in more favourable long-term physical health outcomes.

## Supplementary material

For supplementary material accompanying this paper visit http://dx.doi.org/10.1017/S0033291719000308. click here to view supplementary material
