# Supplementary material for: Insulin resistance and obesity, and their association with depression in relatively young people: findings from a large UK birth cohort
Source: Psychol Med. 2019 Mar 11;50(4):556–65. doi: 10.1017/S0033291719000308 (PMC7093318; doi:10.1017/S0033291719000308)
Supplement: Supplementary file 1 [file S0033291719000308sup001.docx]

**Insulin Resistance and Obesity and their Association with Depression in Relatively Young People: Findings from a Large U.K. Birth Cohort**

Perry, B.I.; Khandaker, G.M.; Marwaha, S.; Thompson, A.; Zammit, S.; Singh, S.P.; Upthegrove, R.

**Supplementary Data**

Supplementary Data Table 1: Complete cases: Cross-sectional association between dysglycaemia/BMI and depressive symptoms (age 18)

| **Predictor** | **Regression Co-efficient (95% C.I.) for Depressive Symptoms** | | | | | | | | | | |
| --- | --- | --- | --- | --- | --- | --- | --- | --- | --- | --- | --- |
|  | **Adjustments** | | | | | | | | | | |
|  | **Unadjusted Model** | | | **Demographic adjustments** (sex, birth social class, ethnicity, maternal education, maternal EPDS score) | | **Lifestyle adjustments** (BMI^1^, HOMA_2_^2^, smoking, alcohol use physical activity) | | **Immune adjustments** (IL-6 (9y), CRP (18y)) | | **Complete model** (all adjustments) | |
| **Depressive Symptoms** |  |  |  |  |  |  |  |  |  |  |  |
|  | **n** | **B (95% C.I.)** | **p** | **B (95% C.I.)** | **p** | **B (95% C.I.)** | **p** | **B (95% C.I.)** | **p** | **B (95% C.I.)** | **p** |
| HOMA_2_ | 2231 | 0.04 (0.01-0.19) | 0.042* | 0.03 (-0.24-0.11) | 0.484 | 0.06 (-0.09-0.22) | 0.438 | 0.04 (0.01-0.18) | 0.039* | 0.01 (-0.06-0.05) | 0.886 |
| FPG | 2231 | -0.05 (-0.13- -0.02) | 0.044* | -0.05 (-0.23-0.12) | 0.540 | -0.05 (-0.21-0.06) | 0.431 | -0.05 (-0.10 - -0.03) | 0.040* | -0.01 (-0.09-0.03) | 0.310 |
| Fasting Insulin | 2231 | 0.05 (0.01-0.09) | 0.012* | 0.03 (-0.18-0.16) | 0.935 | 0.04 (-0.04-0.27) | 0.153 | 0.05 (0.02-0.09) | 0018* | 0.01 (-0.03-0.10) | 0.993 |
| BMI | 2231 | 0.04 (0.02-0.07) | 0.045* | 0.03 (-0.02-0.07) | 0.252 | 0.03 (-0.02-0.67) | 0.218 | 0.04 (0.04-0.10) | 0.049* | 0.03 (-0.04-0.04) | 0.158 |
| ^1^Not adjusted for in BMI analysis ^2^Not adjusted for in HOMA/FPG/FI analysis  *Indicates p<0.05 | | | | | | | | | | | |

Supplementary Data Table 2: Complete cases: Cross-sectional associations between dysglycaemia/BMI and depressive episode (age 18 years)

| **Predictor** | |  | **Odds Ratio (95% C.I.) for Depressive Episode** | | | | | | | | |  | |
| --- | --- | --- | --- | --- | --- | --- | --- | --- | --- | --- | --- | --- | --- |
|  | |  | **Adjustments** | | | | | | | | |  | |
|  |  |  | **Unadjusted Model** | | | **Demographic adjustments** (sex, birth social class, ethnicity, maternal education, maternal EPDS score) | | **Lifestyle adjustments** (BMI^1^, HOMA_2_^2^, smoking, alcohol use, physical activity) | | **Immune adjustments** (IL-6 (9y), CRP (18y)) | | **Complete model**  (all adjustments) | |
| **Depressive Episode** | |  |  |  |  |  |  |  |  |  |  |  |  |
|  | | **n outcome** | **n** | **OR (95% C.I.)** | **p** | **OR (95% C.I.)** | **p** | **OR (95% C.I.)** | **p** | **OR (95% C.I.)** | **p** | **OR (95% C.I.)** | **p** |
| HOMA_2_ | | 179 | 2231 | 1.26 (1.09-1.43) | 0.023* | 1.01 (0.84-1.23) | 0.913 | 1.06 (0.91-1.24) | 0.341 | 1.25 (1.10-1.40) | 0.029* | 1.02 (0.64-1.61) | 0.943 |
| FPG | | 179 | 2231 | 0.83 (0.56-1.23) | 0.353 | 0.79 (0.50-1.28) | 0.426 | 0.89 (0.76-1.04) | 0.152 | 0.84 (0.50-1.20) | 0.440 | 0.80 (0.49-1.29) | 0.355 |
| Fasting Insulin | | 179 | 2231 | 1.28 (1.10-1.45) | 0.045* | 1.05 (0.87-1.26) | 0.644 | 1.10 (0.94-1.28) | 0.223 | 1.29 (1.09-1.47) | 0.049* | 1.05 (0.66-1.66) | 0.837 |
| BMI | | 179 | 2231 | 1.04 (0.95-1.13) | 0.327 | 1.03 (0.98-1.07) | 0.278 | 1.01 (0.97-1.06) | 0.502 | 1.03 (0.96-1.14) | 0.401 | 1.02 (0.97-.13) | 0.573 |
|  | ^1^Not adjusted for in BMI analysis ^2^Not adjusted for in HOMA/FPG/FI analysis  *Indicates p<0.05 | | | | | | | | | | | | |
